# Using Demand Analysis to Examine Private Practice Mental Health Providers’ Decision to Accept Health Insurance

**DOI:** 10.1007/s10488-026-01507-9

**Published:** 2026-04-28

**Authors:** Kyler R. Lehrbach, Briana S. Last, Elizabeth Rawa, Daniel Zweben, Danielle R. Adams, Donald A. Hantula, Aden Littlewood, Philip C. Kendall, Margaret E. Crane

**Affiliations:** 1 Department of Psychology, Temple University, Philadelphia, USA; 2 Department of Psychology, Stony Brook University, Stony Brook, USA; 3 School of Social Work, University of Missouri, Columbia, USA; 4 Department of Psychiatry and Human Behavior, Warren Alpert Medical School, Brown University, Providence, USA

## Abstract

Less than half of insured Americans can access mental health care due to a variety of barriers, including low provider participation in insurance networks. Providers report insufficient reimbursement rates and administrative burdens as reasons they do not accept insurance. We examined the importance of these barriers and the extent to which reimbursement rates would need to increase for private practice providers to accept insurance. Private practice mental health providers (i.e., psychologists, marriage and family therapists, mental health counselors, and social workers) completed an online survey containing two behavioral economic tasks. In the *Criteria Ranking Task*, providers (*n* = 326) ranked barriers that most deterred them from accepting insurance, reporting insufficient reimbursement rates as the most important, followed by administrative burdens. In the *Hypothetical Demand Task*, providers indicated the likelihood of accepting Medicare at various reimbursement rate increases with and without hypothetical administrative assistance. Participants indicated they would accept insurance following 83% rate increases (P_max_ = $188.97) for doctoral providers (*n* = 188) and 118% rate increases (P_max_ = $168.51) for master’s-level providers (*n* = 112). If provided administrative assistance, participants indicated they would accept insurance following 100% rate increases (P_max_ = $206.28) for doctoral providers and 169% rate increases (P_max_ = $207.90) for master’s-level providers. Results support existing research findings that insufficient reimbursement rates and administrative burdens are the primary barriers to mental health providers accepting insurance. Policy efforts to increase access to mental healthcare must consider the incentives and burdens that impact providers and their decision to accept insurance.

**Supplementary Information:**

The online version contains supplementary material available at 10.1007/s10488-026-01507-9.

## Introduction

Approximately half of American adults with a diagnosed mental health disorder do not seek care because they cannot afford it (SAMHSA, 2021). When they do seek care, 57% cannot access treatment (Sky et al., 2023). Commonly reported access barriers include difficulty accessing in-network providers and long waitlists (Andrade et al., 2014). The vast majority (i.e., 65–70%) of mental health providers (e.g., psychologists, social workers, mental health counselors) work in private practice, which refers to solo or group settings that providers own and operate separately from hospitals, medical facilities, healthcare organizations, human service organizations, or government agencies (American Psychological Association, 2024; Lombardi et al., 2022). Although some private practice providers accept commercial or public (e.g., Medicare, Medicaid) insurance, many do not have the administrative support to manage insurance billing and claims, and surveys consistently find that around a third of private practice providers do not accept any form of insurance (American Psychological Association, 2024; Bishop et al., 2014; Zencare Group, Inc., 2022; Zhu et al., 2024). The rates of private practice providers that accept Medicaid, which typically reimburses lower than commercial insurance or Medicare, is even lower (Wen et al., 2019). Thus, many patients must pay for the costs of therapy fully via self-pay or out-of-pocket when accessing care through private practice providers (Gao & Olfson, 2025). These self-pay rates can be prohibitive for some patients, with recent analyses finding that average individual psychotherapy session fees were $157 (Centers for Medicare and Medicaid Services, 2024; Heard Technologies Inc., 2024). Considering that mental health sessions typically happen weekly, this expense adds up quickly and is cost-prohibitive for most individuals. Mental health treatment-seekers who have no choice but to self-pay for services may experience an increase in financial stress, meaning treatment itself can result in negative mental health outcomes (Bialowolski et al., 2021; Butterworth et al., 2009; McMorrow et al., 2017).

Private practice mental health providers frequently report that they do not accept insurance because of low reimbursement rates; they can often make far more money—even double—via self-pay (Albizu-García et al., 2004; American Psychological Association, 2024; Centers for Medicare and Medicaid Services, 2024; Hamp et al., 2016; Heard Technologies Inc., 2024; Regier et al., 2008; Zencare Group, Inc., 2022). Of note, mental health providers holding doctoral degrees (e.g., clinical psychologists with PsyDs or PhDs) are reimbursed at 100% of the Medicare rate while master’s-level mental health providers (e.g., social workers, mental health counselors) are reimbursed at 75% of the Medicare rate (Centers for Medicare and Medicaid Services, 2024; Heard Technologies Inc., 2024). Because commercial insurance rates are proprietary and negotiated, it is challenging to identify reimbursement rates. Medicare rates, which are publicly available, are important indicators as they influence the rates used by state Medicaid and commercial insurance (Bailey, 2020). Notably, Medicare reimbursement rates for a 45-minute psychotherapy session have only increased 2.5% ($100.62 to $103.20) from 2001 to 2024, despite inflation rising 75% in that timeframe, suggesting that insurance reimbursement rates for mental health care may not be keeping pace with the rising costs of living (Centers for Medicare and Medicaid Services, 2024; Medicare Learning Network, 2021; U.S. Bureau of Labor Statistic, 2024).

In addition to insufficient reimbursement rates, mental health providers also report administrative burdens (e.g., billing, submitting/managing claims, technology management), concerns about payment reliability (including prepayment audits and delays), denials of necessary and appropriate care, difficulties paneling/credentialing, and “clawbacks” of previously paid claims as top barriers to accepting insurance (American Psychological Association, 2024; Dunn et al., 2024; Frank et al., 2022; Hamp et al., 2016; Waldman et al., 2024; Zhu & Eisenberg, 2024). One recent investigative journalistic report found that following an allegation of occasional inappropriate billing procedures, an insurer retaliated against an entire practice by clawing back (i.e., requiring a payback of) nearly $11,000 they already paid a provider, and declining to pay them for $1.5 million worth of already delivered services (Waldman et al., 2024). In addition to losing money through payment denials, mental health providers lose time, and, therefore, potential income, handling these administrative burdens, along with increased burnout and attrition associated with these burdens (Zhu & Eisenberg, 2024). Altogether, providers that accept insurance may face double financial penalty through (1) lower reimbursement rates; and (2) lost income due to insurance payment denials, delays, and time dedicated to administrative work. Thus, increases in insurance reimbursement rates or administrative burden relief may financially incentivize providers to participate in insurance networks. Further, intervening on one of these barriers may be enough to offset the financial penalties of the other barrier.

Despite providers’ reports that they do not accept insurance due to low reimbursement rates and insurance-related administrative burdens, there is no systematic evidence suggesting that increasing reimbursement rates or decreasing administrative burdens would increase mental health provider participation in insurance networks (American Psychological Association, 2024). In the present study, we conducted an online, national, cross-sectional survey of private practice mental health providers and used innovative surveying techniques from the field of behavioral economics (an interdisciplinary field dedicated to understanding judgment and decision-making) to examine: (1) providers’ ranked priorities regarding their decision to not accept health insurance for therapy services and (2) providers’ perceived impact of increased reimbursement rates and minimized administrative burdens on their decisions to accept Medicare. Mental health providers indicated the likelihood of accepting Medicare at various reimbursement rate increases with and without hypothetical administrative assistance. It was hypothesized that mental health providers would decide to accept insurance at a lower rate in the context of subsidized administrative assistance relative to when they were asked to indicate their rate with no description of whether administrative assistance would be provided. This was hypothesized due to an assumption that decreasing administrative burdens would be cost saving and therefore account for a portion of reimbursement rate increases desired, consistent with reports in the literature of providers losing potential income due to administrative burdens (Zhu & Eisenberg, 2024).

## Method

All study procedures were approved by the Temple University Institutional Review Board.

### Participants and Procedures

To be eligible for the study, participants had to be licensed mental health providers (i.e., psychologists, marriage and family therapists, mental health counselors, and social workers) who provided individual psychotherapy services in a private practice setting, saw at minimum 10 h of clients per week, and did not accept insurance for at least 50% of their caseload. Psychiatrists and nurse practitioners were excluded from sampling as reimbursement rates for psychotherapy services for these providers are at different rates than the presently studied licensed mental health providers, and previous research has largely centered these medical providers (Centers for Medicare and Medicaid Services, 2024; Zhu & Eisenberg, 2024).

Participants were recruited using email listservs (with permission) for state and national agencies for mental health providers, regional/state-level Facebook groups for mental health providers, and by individual emails sent to contacts obtained using the “APA Psychologist Locator.” The first 200 participants (the recruitment target) who completed the survey received a $5 gift card for participating. Of note, there was an influx of participants following contacting psychologists using the APA Psychologist Locator, resulting in a final sample of 326 mental health providers. A flow diagram detailing recruitment and retainment is provided in the Supplement.

Participants completed a REDCap survey which began with an eligibility screener, followed by a consent form for eligible participants (Harris et al., 2019). Surveys were completed by participants between October 2024 and January 2025. Consented participants then completed the *Criteria Ranking Task*, the *Hypothetical Demand Tasks*, and demographic questions.

### Measures

The administered survey, including all study measures, is included in the Supplement. The *Criteria Ranking Task* is on pages 4–6 of the survey, and the *Hypothetical Demand Task* is on pages 11–33 of the survey.

#### Criteria Ranking Task

Participants first completed a *Criteria Ranking Task*, an investigator-created ordinal ranking exercise which prompted participants to rank six barriers to accepting insurance from most important (i.e., 6) to least important (i.e., 1): “reimbursement rates are too low,” “administrative burden of billing/submitting claims,” “delays in receiving payment,” “concerns about treatment regulations,” “inability to receive acceptance onto an insurance panel,” and “other,” which served as a free response option. These barriers were selected based on a literature review of mental health providers’ reported barriers to insurance acceptance (American Psychological Association, 2024; Dunn et al., 2024; Frank et al., 2022; Hamp et al., 2016; Waldman et al., 2024; Zhu & Eisenberg, 2024). Insufficient reimbursement rates (particularly compared to self-pay options) are established as relevant to mental health provider decision making to accept insurance (Albizu-García et al., 2004; American Psychological Association, 2024; Centers for Medicare and Medicaid Services, 2024; Hamp et al., 2016; Heard Technologies Inc., 2024; Regier et al., 2008; Zencare Group, Inc., 2022). Administrative burdens of billing/claims, payment delays, and concerns about treatment regulations are also identified as barriers in previous research (American Psychological Association, 2024; Dunn et al., 2024; Frank et al., 2022; Hamp et al., 2016; Waldman et al., 2024; Zhu & Eisenberg, 2024). These factors have financial impacts, and their inclusion in the *Criteria Ranking Task* allowed direct comparison of these barriers with insufficient reimbursement rates. We included the “inability to receive acceptance onto an insurance panel” to account for a structural barrier that might impact insurance participation above and beyond the aforementioned factors (American Psychological Association, 2024). Finally, including the “other” option provided the opportunity for participants to specify factors presently overlooked in the literature.

This measure used a ranking exercise to force participants to identify which of the barriers were most important to insurance acceptance. This was used instead of a rating exercise (e.g., Likert scale) to avoid central tendency responding or “ceiling effects” where all barriers could have been rated as “most important” (Hessling et al., 2004; Kusmaryono et al., 2022). We sought to identify the most important barriers to insurance acceptance to streamline policy implications for maximum impact. Additionally, research has found that rank-based questionnaires are more methodologically sound and consistent than rating-based questionnaires, curbing some limitations of subjective reporting (Yannakakis & Hallam, 2011; Yannakakis & Martínez, 2015). Previous health-related studies have used ordinal ranking exercises (Ali & Ronaldson, 2012). Participants indicated their eligibility to accept different insurances based on the populations they served and then completed the *Criteria Ranking Task* for those insurances; all 326 participants completed the task for commercial insurance, 312 participants completed the task for Medicaid (as participants who only work with populations eligible for Medicare coverage were excluded), and 134 participants completed the task for Medicare (as the remainder of participants did not indicate working with populations eligible for Medicare).

#### Hypothetical Demand Task

Participants were next asked to complete the *Hypothetical Demand Tasks*. *Hypothetical Demand Tasks* are a kind of demand analysis, a widely used technique from behavioral economics that examines how consumption or use of a commodity changes as its cost increases or decreases (Hursh, 1980). Hypothetical demand tasks have been used to assess the likelihood of engaging in behaviors such as therapy treatment selection, medication adherence, service pricing, and occupational decisions (Gilroy et al., 2022; Gilroy & Feck, 2022; Hayashi et al., 2022; Hursh, 1980, 1984; Jarmolowicz et al., 2019, 2020; Malkin et al., 2023). The investigator-created hypothetical demand task in the present study examined the likelihood of a mental health provider accepting insurance (consumption) relative to reimbursement rate (cost), generating a demand curve representing elasticity, defined as the likelihood of accepting insurance in response to reimbursement rate changes. We also explored whether insurance acceptance was responsive to changes in administrative burdens.

In the *Hypothetical Demand Task*, participants were prompted to rate their likelihood of accepting insurance (on a scale of 0 [not likely] to 100 [likely]) on twelve trials of various Medicare reimbursement amounts, beginning with their current Medicare reimbursement amount (as determined by their reported zip code, per publicly available data from the CMS website; Centers for Medicare and Medicaid Services, 2024). Only acceptance of Medicare was examined for the *Hypothetical Demand Task* as Medicaid and commercial insurance rates are not publicly available, preventing accurate rate increase estimation. Participants were told to imagine that they are providing services for a client who is insured under Medicare, that they are accepted on the insurance panel for Medicare, that it functions identically to current circumstances besides reimbursement rate, and that they are providing a 45-minute psychotherapy session. The subsequent eleven trials increased the reimbursement rate at the various percentage raises of their Medicare reimbursement rate. For example, someone who would be reimbursed at the national payment amount of $103 (i.e., unadjusted for geographic location) received trials of the following raises: 10% ($113), 25% ($129), 50% ($155), 75% ($180), 100% ($206), 125% ($232), 150% ($257), 175% ($283), 200% ($309), 250% ($360), and 300% ($412). The following instructions were presented at each trial (where X represents the Medicare reimbursement amount for that trial):

*If you were reimbursed X by Medicare*,* how likely would you be to accept their health insurance? (On a scale of 0 [not likely] to 10 [likely])?*

The same rates were presented for both doctoral and masters-level participants, although doctoral participants (i.e., psychologists) are reimbursed at 100% of the Medicare reimbursement rate, while masters-level participants (i.e., social workers, mental health counselors, and marriage and family therapists) are reimbursed at 75% of the Medicare reimbursement rate (Centers for Medicare and Medicaid Services, 2023). For this reason, doctoral (*n* = 188) and master’s-level (*n* = 112) providers’ data were analyzed separately.

After the first series of twelve trials, another series of twelve trials were presented in the same manner, except participants were instructed to answer under the additional condition that administrative assistance was subsidized (i.e., thus relieving administrative burden). The following instruction was presented before the second set of trials:

*For the following questions*,* please indicate how likely you would be to accept Medicare insurance based on different reimbursement rates*,* with the added assumption that additional administrative burdens of accepting insurance are handled by a third party*,* at no cost to you as a provider.*

#### Demographics

Participants completed background questions, including race, gender identity, and licensure type. Participants also reported their current billing rate, their self-pay fees, the zip code they live in (to determine cost-of-living/relevant geographic insurance data), and the state where they primarily provide therapy services. Participants also provided their national provider identifier (NPI) (a publicly available identifier), which was used to verify their status as a licensed provider.

### Analytic Plan

Study analyses included descriptive statistics for participant demographics and the *Criteria Ranking Task*.

#### Dependent Measure and Other Indices of Demand

The primary outcome measure for the *Hypothetical Demand Task*, analytic P_max_, represents the point where demand shifts from inelastic (insensitive to a change in price) to elastic (relatively sensitive to a change in price), indicating the point at which mental health providers’ preference shifts from rejecting insurance to accepting insurance (Gilroy et al., 2019). In the present analyses, P_max_ can be directly interpreted as the reimbursement rate at which a provider’s preference shifts to consider accepting insurance. Several additional indices of demand analysis provide insight into understanding the demand curve and were analyzed for the current study and reported in the supplement as sensitivity analyses.

Nonsystematic responding was excluded from data using established criteria: trend (i.e., consumption not reducing by at least a 0.025 log-unit range from the first to last reimbursement rate; 4 cases total), bounce (i.e., excessive “jumps” in consumption that exceed 25% of initial consumption at lowest price; 0 cases total), or reversal from zero (i.e., consumption is resumed at a higher price after consumption has already ceased, or in the case of this study, insurance is not fully accepted at a higher reimbursement rate after fully accepted at a lower rate; 5 cases total). In line with previous research, the trend criterion was relaxed such that data was not excluded which indicated the choice of never accepting insurance (i.e., exclusively choosing 0 as the likelihood of acceptance; 9 cases) or the choice of always accepting insurance (i.e., exclusively choosing 10 as the likelihood of acceptance; 15 cases), as these may be deliberate responses that are relevant for the research question (Hayashi et al., 2022; Stein et al., 2015).

To adhere to the law of demand (i.e., consumption decreases as cost increases), participant scores were reverse-scored such that likelihood to *reject* insurance decreased as reimbursement rates increased (Samuelson & Nordhaus, 1985). This reverse-scoring does not impact P_max_, as the point at which preference shifts from rejecting insurance to accepting insurance remains the same. An online application, *shinybeez*, was used to produce the demand analysis model (Kaplan and Reed 2025a). *Shinybeez* is a user-friendly application available through a web browser that utilizes the R package *beezdemand* (Kaplan et al., 2018). Following initial demand analyses, post-hoc analyses of a Wilcoxon signed-rank test in R were used within groups to determine statistical significance between P_max_ of providers responding on the first set of trials compared to the second set of trials (i.e., with and without subsidized administrative assistance).

## Results

Participant demographics are provided in Table 1. The total sample of participants (*n* = 326) was 83% white, 80% female, and 63% doctoral-level providers. Following completion of the Criteria Ranking Task, 17 participants discontinued prior to or during the Hypothetical Demand Task, and 9 participants were excluded per having nonsystematic data per the description in the analytic plan. Demographic breakdown in the Hypothetical Demand Task (83% white, 80% female, and 63% doctoral-level provider) did not differ compared to the total sample.

**Table 1:** Clinician demographics from total sample of usable data

Characteristic	*N*	%
*Race/Ethnicity*		
White/European American	270	82.8%
Latino/a/x	13	4.0%
Asian or Asian American	12	3.7%
Bi/Multiracial	7	2.1%
Black or African American	6	1.8%
Arab American, Middle Eastern, or North African	5	1.5%
American Indian, Alaska Native, and/or Indigenous	3	0.9%
Another race or ethnicity not listed above	1	0.3%
Prefer not to answer	9	2.8%
*Gender Identity*		
Female/Woman	262	80.4%
Male/Man	53	16.3%
Nonbinary	6	1.8%
Prefer not to answer	5	1.5%
*Transgender Identification*		
No	310	95.1%
Yes	11	3.4%
Prefer not to answer	5	1.5%
*License Type*		
PhD, PsyD	204	62.6%
LCSW, LMSW, LICSW	80	24.5%
LPC	21	6.4%
LMHC	13	4.0%
LMFT	8	2.5%
*Practice Context*		
Solo practice	270	82.8%
Group practice	56	17.2%
*Service Area*		
Urban	169	51.8%
Suburban	141	43.3%
Rural	16	4.9%
*Sliding Scale Offered*		
Yes	212	65.0%
No	114	35.0%
*Financial Characteristic*		
Self-Pay Fee	$211.72 (mean)	$200.00 (median)
Lowest Sliding Scale Fee	$87.91 (mean)	$85.00 (median)
Estimated Annual Income	$134,948.28 (mean)	$115,000.00 (median)

### Criteria Ranking Task

Table 2 shows descriptive statistics of reported rankings of barriers for not accepting Medicare, Medicaid, or commercial insurance for surveyed mental health providers. Across all three types of insurance, insufficient reimbursement rates were ranked as the most important barrier to accepting insurance and the administrative burden of submitting claims were ranked as the second most important barrier. Providers ranked “concerns about treatment regulations” slightly higher as a reason to not accept Medicare compared to their rankings for not accepting Medicaid or commercial insurance.

**Table 2:** Ranking of barriers to accepting insurance

Insurer	Reason	Mean	SD
Medicare			
	Reimbursement rates are too low	2.54	1.79
	Administrative burden of submitting claims	2.77	1.54
	Concerns about treatment regulations	3.40	1.22
	Delays in getting paid	3.61	1.07
	Concerns about audit risk	3.76	1.37
	Not accepted onto an insurance panel	4.92	2.00
Medicaid			
	Reimbursement rates are too low	2.08	1.77
	Administrative burden of submitting claims	2.70	1.45
	Delays in getting paid	3.44	1.04
	Concerns about treatment regulations	3.59	1.20
	Concerns about audit risk	3.96	1.28
	Not accepted onto an insurance panel	5.04	1.85
Commercial Insurance			
	Reimbursement rates are too low	2.24	1.82
	Administrative burden of submitting claims	2.87	1.48
	Delays in getting paid	3.44	1.12
	Concerns about treatment regulations	3.48	1.17
	Concerns about audit risk	3.87	1.28
	Not accepted onto an insurance panel	5.11	1.75

Participants also had the opportunity to provide other reasons not captured if they felt that would be a part of their rankings. 79 participants indicated that “other” would be a part of their rankings. 33 of these responses were expanding on specific scenarios related to barriers already captured in the ranking (e.g., detailing payment delays). The remaining 46 responses included a variety of barriers which other participants may have categorized under “administrative burden of submitting claims,” most commonly naming “clawbacks of payment.” Multiple participants also mentioned client privacy/confidentiality and feeling not valued by insurance companies as meaningful barriers. Several participants also expressed their desire to rank every barrier as most important, with one participant noting “All of the above – the juice ain’t worth the squeeze.”

### Hypothetical Demand Task

**Fig. 1:**
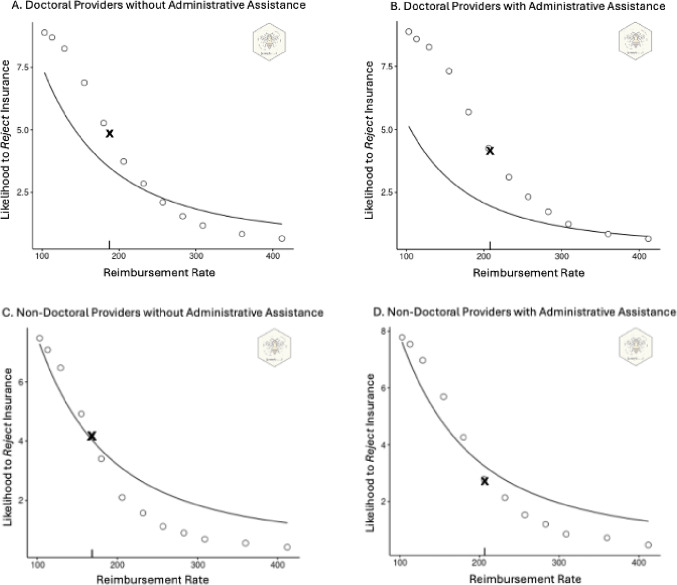
Demand curves of likelihood of rejecting insurance *Note.* Y-axis begins at the current reimbursement rate. The “x” on each plot signifies location of P_max_

Figure 1 shows the demand curve for the mean of each group and condition, with P_max_ (i.e., the reimbursement rate at which a provider’s preference shifts to consider accepting insurance) plotted on each graph. Across all demand curves, likelihood of rejecting insurance decreased as reimbursement rate increased (i.e., likelihood of accepting insurance increased as reimbursement rate increased). Doctoral-level providers responding to exclusive increases in reimbursement rates had a P_max_ of $188.97, indicating these providers would need an 83% increase in reimbursement rates (relative to the Medicare national average) for their preference to shift to accepting insurance. When prompted to respond to the same task with the addition of subsidized administrative assistance to decrease burden, P_max_ increased to $206.28, or a 100% increase in reimbursement rate relative to the Medicare national average. A Wilcoxon signed rank test indicated that the difference between the P_max_’s of $188.97 and $206.28 was statistically significant (W = 3460.5, p = < 0.05). A Hodges-Lehmann estimator indicated that the P_max_’s of the trial with subsidized administrative assistance were typically higher than the P_max_’s of the first trial by $13.50 ($$\:\widehat{\theta}$$= 13.50, 95% CI [0.00, 25.19]).

Master’s-level providers responding to exclusive increases in reimbursement rates had a P_max_ of $168.51, indicating they would need an 118% increase in reimbursement rates (relative to the Medicare national average) for their preference to shift to accepting insurance. When prompted to respond to the same task with the addition of subsidized administrative assistance to decrease burden, P_max_ shifted to $207.90, or a 169% increase in reimbursement rate relative to the Medicare national average. A Wilcoxon signed rank test indicated that the difference between the P_max_’s of $168.51 and $207.90 was statistically significant (W = 1877, p = < 0.01). A Hodges-Lehmann estimator indicated that the P_max_’s of the trial with subsidized administrative assistance were typically higher than the P_max_’s of the first trial by $20.99 ($$\:\widehat{\theta}$$= 20.99, 95% CI [7.99, 30.99]). Doctoral providers charged an average of $234.18 per session while masters-level providers charged an average of $175.39, both of which are greater than their respective P_max_ from the *Hypothetical Demand Task*. Sensitivity analyses of other indices of demand, as provided in the Supplemental material, are consistent with the primary analyses.

## Discussion

The present findings demonstrated how current reimbursement rates within the health insurance system deter mental health providers from accepting insurance for therapy services. Consistent with prior findings, our survey’s participants indicated that insufficient reimbursement rates are the top barrier to mental health providers’ acceptance of insurance (American Psychological Association, 2024). Providers’ responses to the hypothetical demand task suggested that increasing reimbursement rates may increase providers’ participation in insurance networks. Also consistent with prior findings, the administrative burden of submitting claims was ranked as the second most important barrier to treatment (American Psychological Association, 2024). Payment delays, treatment autonomy, concerns about audit risk, and difficulties with insurance paneling were relevant barriers to accepting insurance, but to a lesser extent. By allowing participants to rank-order their most important barriers, results provided clarity as to which barriers policy efforts should target for maximal impact.

Using a behavioral economic framework, findings illustrated how much reimbursement rates would hypothetically need to increase to incentivize mental health providers to accept insurance. Across all mental health providers, willingness to accept insurance increased as reimbursement rates increased (i.e., the likelihood of rejecting insurance decreased as rates increased). Results demonstrated that an increase in reimbursement would impact the decision-making of mental health providers to accept insurance at differing rates between doctoral and master’s-level providers. Specifically, doctoral providers would need an 83% increase in reimbursement rate (P_max_ = $188.97) and master’s level providers would need a 118% increase in rate (P_max_ = $168.51) to accept insurance. These findings illustrate that current Medicare reimbursement rates (with a national payment amount of $103) are considerably lower than what providers perceive to be appropriate for the care they deliver. For example, North Carolina, an area with an extreme shortage in behavioral health workforce, has a Medicare reimbursement rate of $100.82 (or $75.62 for masters-level providers) (Centers for Medicare and Medicaid Services, 2024; Rural Health Information Hub, n.d.). Our results suggest that increasing the Medicare reimbursement rate in North Carolina by 87% (to the P_max_ of $188.97) may motivate doctoral-level providers to accept insurance, while increasing the Medicare reimbursement rate in North Carolina by 128% (to the P_max_ of $168.51) may motivate doctoral-level providers to accept insurance.

Our exploratory analyses, evaluating the preference for accepting insurance with the inclusion of subsidized administrative assistance, yielded unexpected findings. Given the potential lost income related to managing administrative burdens, we originally hypothesized that providers would need rates to increase less if administrative assistance was provided to them at no cost. On the contrary, providers indicated that they would need even greater rate increases to accept health insurance under the condition of subsidized administrative assistance, with doctoral providers needing a 100% raise (P_max_ = 206.28) and master’s level providers needing a 169% raise (P_max_ = 207.90). There are several potential explanations for this unanticipated finding. First, it is possible that our mention of administrative burdens in the task instructions of the second hypothetical scenario may have reminded providers of the financial and time costs of managing administrative work associated with insurance acceptance, which may not have factored into their calculations in the original hypothetical scenario. Second, providers may have interpreted the task instructions as indicating that there would be more administrative burdens in the second hypothetical scenario than those in the original hypothetical scenario. If providers interpreted the instructions in this light, their responses that rates would have to increase more to compensate for these perceived additional burdens would, in fact, be consistent with our hypothesis that providers experience administrative burdens as financially costly. Given potential shortcomings regarding how the task introduced the concept of subsidized administrative assistance, such as a lack of clarity regarding how that would take shape, it is unclear which of these explanations account for the unexpected findings. Altogether, while unanticipated, the results from the *Hypothetical Demand Task* with the addition of subsidized administrative assistance may underscore mental health providers’ perceived burdens of administrative tasks within the insurance system, consistent with findings from recent in-depth investigative reporting on mental health providers who left insurance networks due to these burdens (Waldman et al., 2024). These results further shine light on the importance of understanding the unique administrative burden that mental health providers face when participating in insurance networks, in line with previous research (Dunn et al., 2024; Zhu & Eisenberg, 2024).

### Limitations

Limitations merit consideration. Primarily, the nature of the hypothetical demand task is indeed hypothetical, such that it is unknown if providers’ real-world decision to accept insurance would actually change at the indicated raises. Thus, it remains to be seen what rate raises would be necessary to instill behavior change and how that might shift over time. Further, based on participants’ questions to the study email account, some participants may have found the survey instructions for the criteria ranking or hypothetical demand tasks unclear. This could have led some participants to respond to the survey in unexpected ways or to merely discontinue the study. In particular, there may have been lack of clarity with what “subsidized administrative assistance” meant on the second trial of the *Hypothetical Demand Task*. A clearer operationalization of what we meant by no-cost administrative assistance, a comprehension check to confirm participants’ understanding of the task instructions, and randomizing the order of the presentation of the trials in the *Hypothetical Demand Task* (i.e., with and without subsidized administrative assistance) would have enabled us to draw firmer conclusions about providers’ responses.

Although our *Criteria Ranking Task* offers useful information about what deters providers from accepting commercial insurance in general, there is also substantial variability in reimbursement rates and administrative burdens between different commercial insurers and Medicaid Managed Care Organizations, which our study did not explicitly examine (Cooper et al., 2019). Attrition, while normal in any study, is an additional limitation, as many participants (*n* = 277) discontinued the study either because they were confused by some of the survey instructions or during the demographic portion of the survey, potentially due to apprehension providing their NPI number (which we used to ensure providers were licensed and to ward against fraudulent participants, common in online research; Bindman, 2013) Finally, our sample (while demographically representative of the field regarding gender, racial, and ethnic diversity) overrepresented doctoral-level psychologists relative to the behavioral health workforce, as a 2021 report indicated that there were only 95,865 doctoral-level psychologists compared to 552,890 social workers, 112,948 mental health counselors, and 26,763 marriage and family therapists providing services in the United States (National Center for Health Workforce Analysis, 2023). This oversampling is noteworthy in research investigating provider-desired rates as doctoral-level providers are able to command higher wages than their masters-level counterparts.

### Future Directions

Despite these limitations, this study is the first to empirically examine the extent to which reimbursement rates and administrative burdens deter mental health providers from participating in insurance networks and determine how much rates need to be increased for providers to switch from not accepting to accepting Medicare. Additionally, this study addresses a gap in the literature on empirical evidence regarding insurance acceptance by masters- and doctoral-level providers, as existing research on insurance acceptance for mental health treatment has focused on psychiatrists (Zhu & Eisenberg, 2024). Future endeavors should continue to explore the decision making of medical providers, including those that were excluded from this study (e.g., psychiatric nurse practitioners, psychiatric physician assistants, psychiatrists), as well as mental health providers whose work extends beyond individual psychotherapy services (e.g., couples psychotherapy, group psychotherapy, assessment services). Further, while our investigations revealed significant elasticity (i.e., amount that change in reimbursement rates result in a change of likelihood to accept insurance) in providers’ decisions to accept insurance, previous research has identified much lower elasticity in mental health service provision when rates increase (Alexandrov et al., 2025). Thus, future research must examine whether providers’ response to the hypothetical demand task are ecologically valid – that is, investigations of how much reimbursement rate increases lead to increased network participation and increased access to mental healthcare are needed.

As for policy, our results confirm growing calls to increase providers’ reimbursement rates to address the shortage of mental health providers accepting health insurance, through Medicare, Medicaid, or commercial insurance options. Our findings also point to the need for regulations on the administrative burdens associated with insurance. Many states and the federal government have implemented regulations, rules, and policies to reduce these burdens, such as “prompt pay” laws requiring insurers to process claims within specific periods of time (Legal and Regulatory Affairs Staff, American Psychological Association, 2005). However, there is some indication that these existing policies are unreliably enforced and several states do not limit insurance companies’ ability to claw back payments (Waldman et al., 2024). Thus, greater regulations and stronger enforcement mechanisms may be needed to actually curb administrative burdens to increase insurance participation. Meaningful policy changes are necessary to increase provider participation in insurance networks to increase the accessibility of mental health services.

## Electronic Supplementary Material

Below is the link to the electronic supplementary material.

Supplementary Material 1

## Data Availability

No datasets were generated or analysed during the current study.
