# Supplementary material for: Using Demand Analysis to Examine Private Practice Mental Health Providers’ Decision to Accept Health Insurance
Source: Adm Policy Ment Health. 2026 Apr 28;53(4):451–61. doi: 10.1007/s10488-026-01507-9 (PMC13372994; doi:10.1007/s10488-026-01507-9)
Supplement: Supplementary file 1 — Supplementary Material 1 [file 10488_2026_1507_MOESM1_ESM.docx]

**Supplementary Material**

**Study Recruitment and Retainment**

The diagram details the number of participants who were ineligible upon screening, eligible and consented, completed the criteria ranking measure, completed demographics, and completed the hypothetical demand task. Data validity was monitored by the study team through various mechanisms including by verifying licensure status using NPI numbers, implementing attention checks, and manually reviewing survey completion time.

Supplemental Figure 1.

*
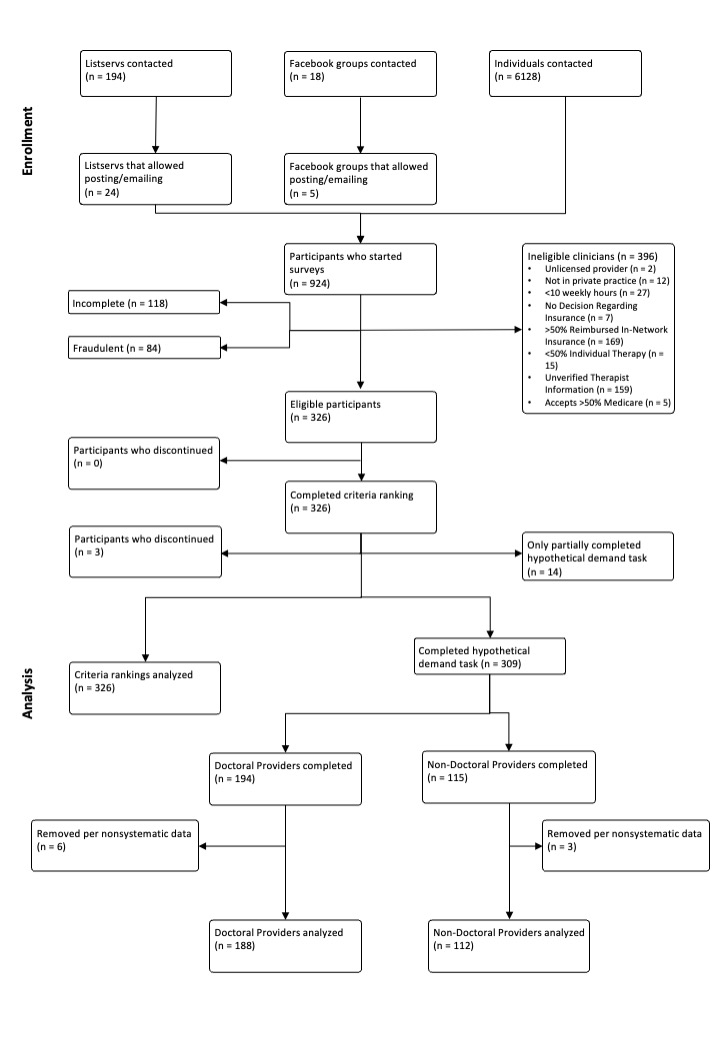
*

**Sensitivity Analyses**

Additional indices of demand analysis include Q_0_ and α. Q_0_ represents demand intensity, or level of consumption when cost is at a very low price, while α represents change in demand elasticity, or sensitivity/ insensitivity of consumption at increases in cost. In our analyses, a lower Q_0_ indicates that providers were more likely to accept insurance at a lower reimbursement rate. A higher α indicates that demand was less elastic (i.e., less sensitive to changes in price).

In the hypothetical demand task, doctoral providers responding to exclusive increases in reimbursement rates had a Pmax of $188.97 (Q0 = 18.58, α = 0.0003). When prompted to respond to the same task with the addition of subsidized administrative assistance to decrease burden, Pmax increased to $206.28 (Q0 = 19.42, α = 0.0002). In the trial without administrative assistance, doctoral providers had a lower Q_0_ and a higher α, indicating that providers were more likely to accept insurance at a lower reimbursement rate and that their demand was less elastic (i.e., sensitive to change in price), respectively, relative to the trial with administrative assistance.

Non-doctoral providers responding to exclusive increases in reimbursement rates had a Pmax of $168.51 (Q0 = 15.3, α = 0.0004). When prompted to respond to the same task with the addition of subsidized administrative assistance to decrease burden, Pmax shifted to $207.90 (Q0 = 16.8, α = 0.0003). In the trial without administrative assistance, non-doctoral providers had a lower Q_0_ and a higher α, indicating that providers were more likely to accept insurance at a lower reimbursement rate and that their demand was less elastic (i.e., sensitive to change in price), respectively, relative to the trial with administrative assistance.

**Survey**

**
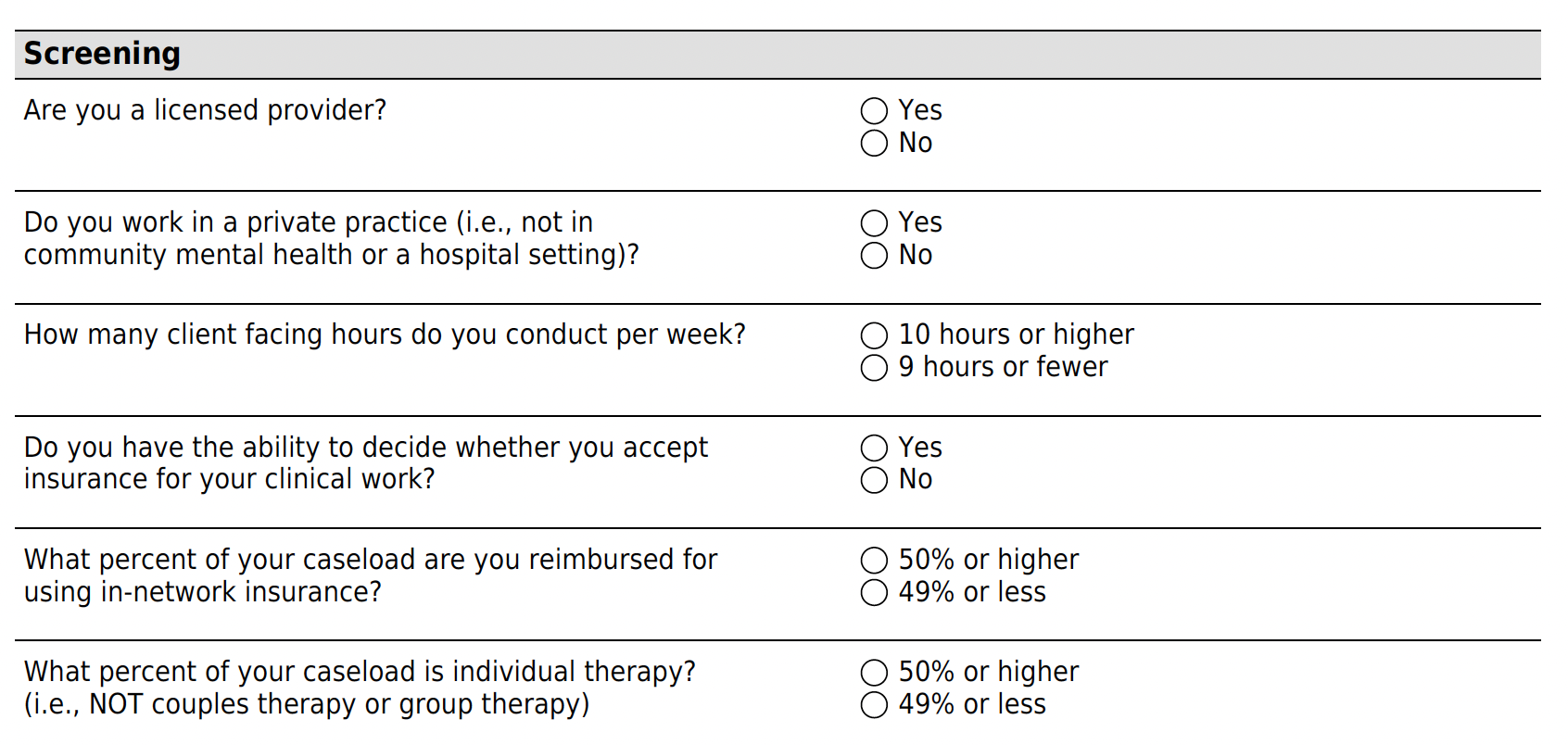
**Page 1

***Discontinues if ineligible***

Page 2


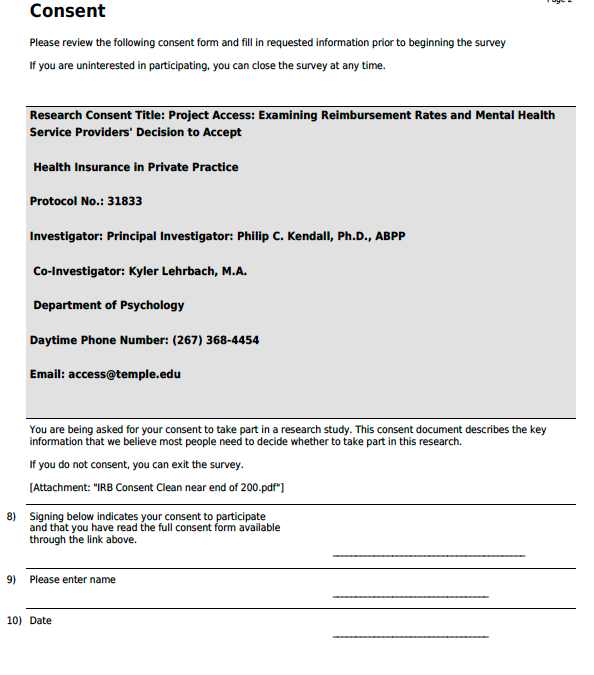


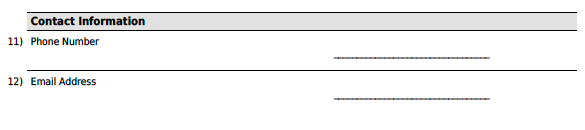

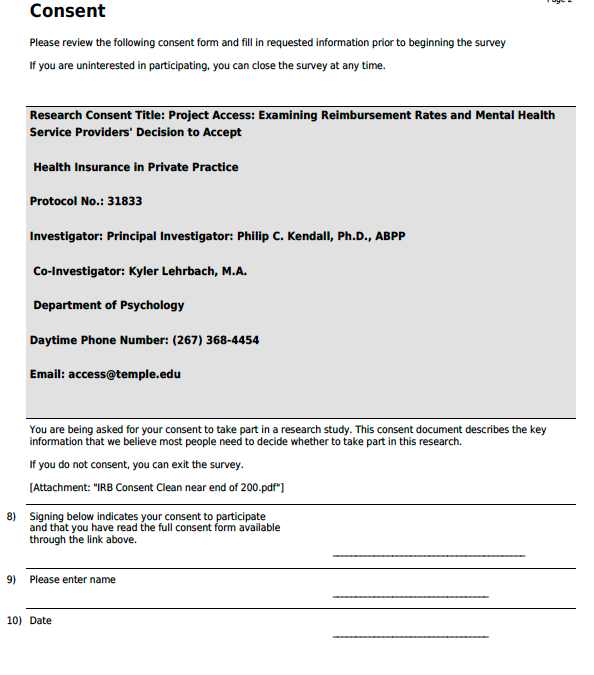


Page 3

**
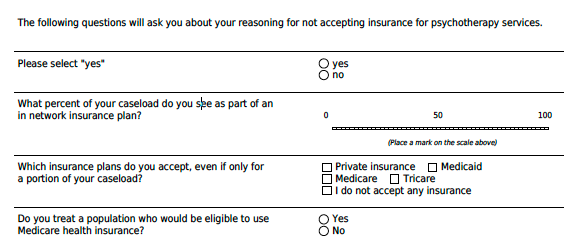
**

**
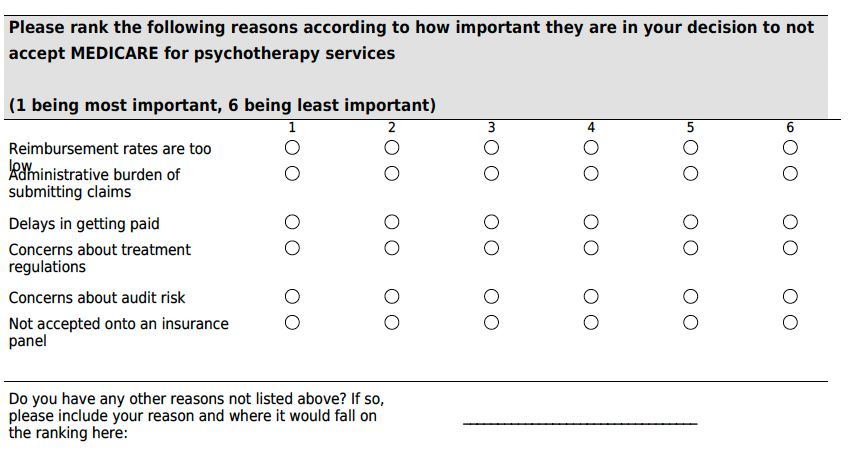
**Page 4

Page 5

**
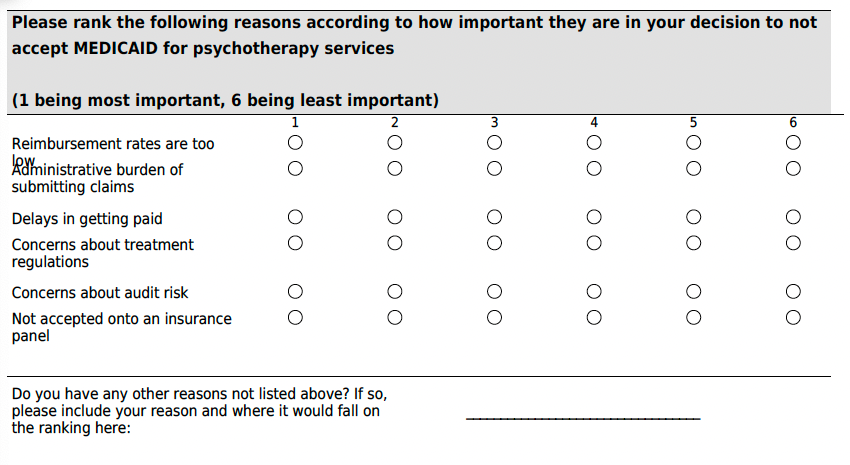
**

Page 6

**
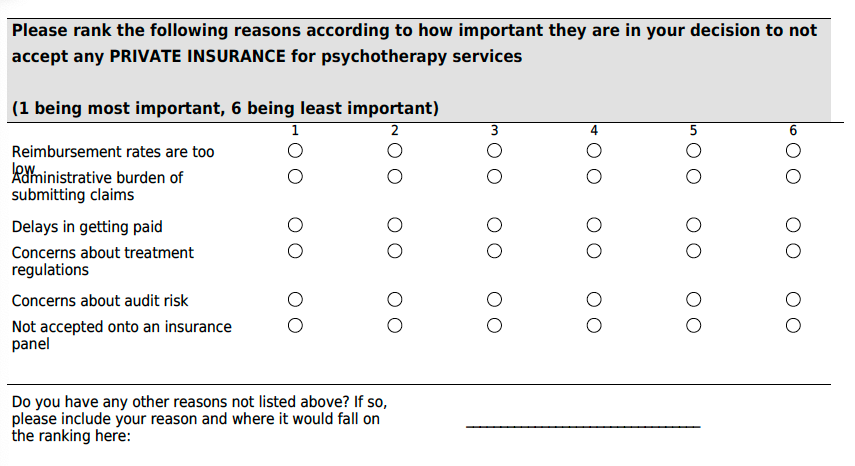
**

Page 7

**
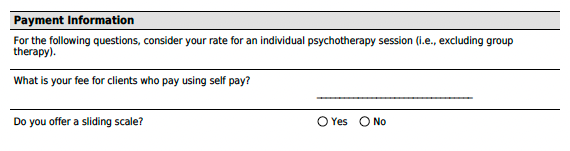
**

Page 8

**
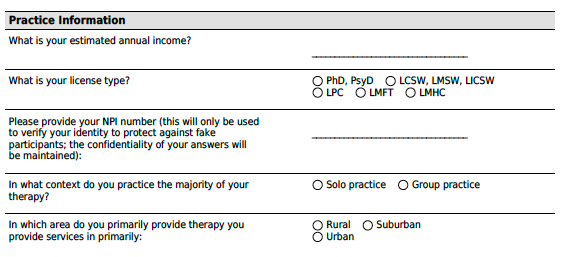
**

Page 9

**
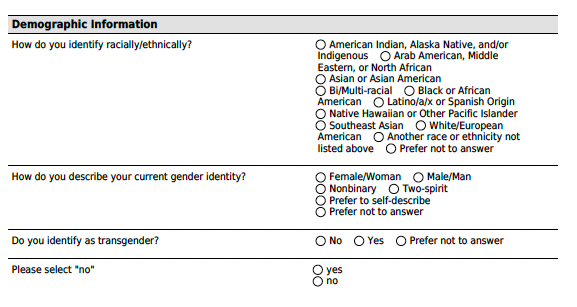
**

**
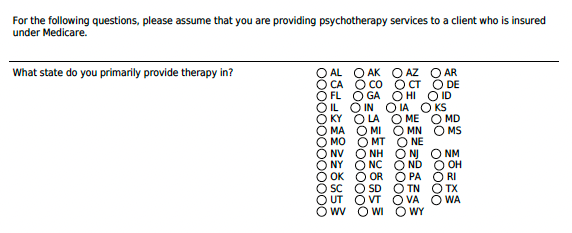
**


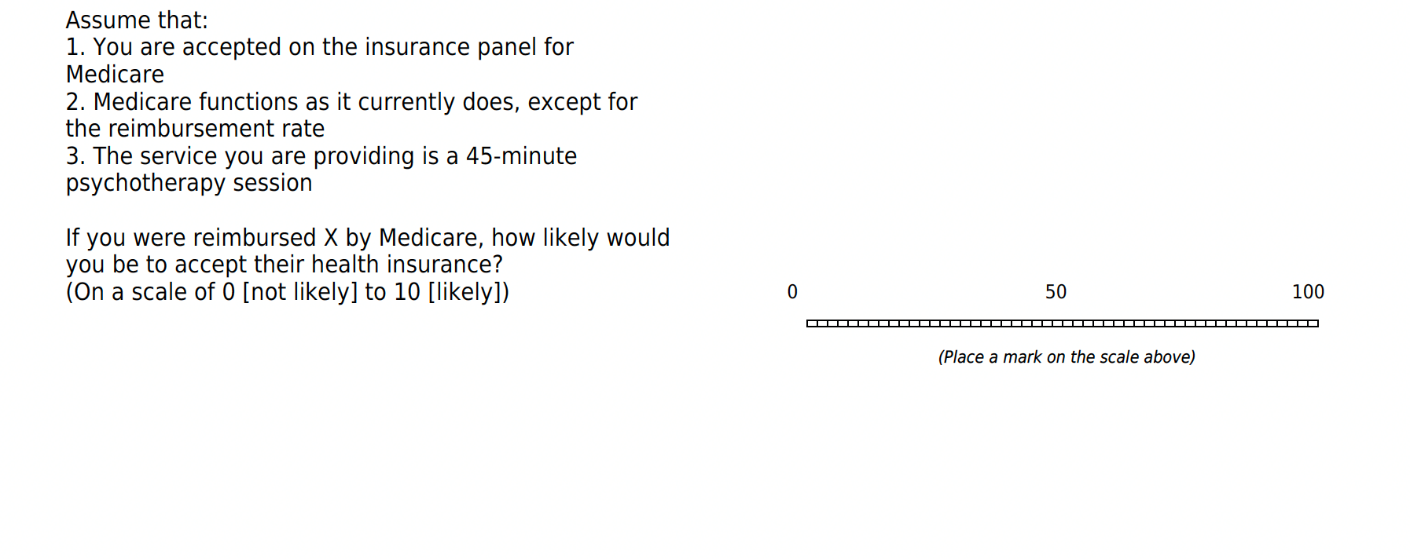
Page 10 (X is their current Medicare rate) **
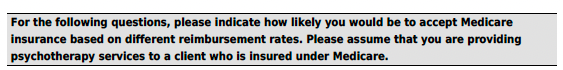
**

*Pages 11-21 are identical to Page 9 but with percentage increases in reimbursement rate as detailed in the methods*


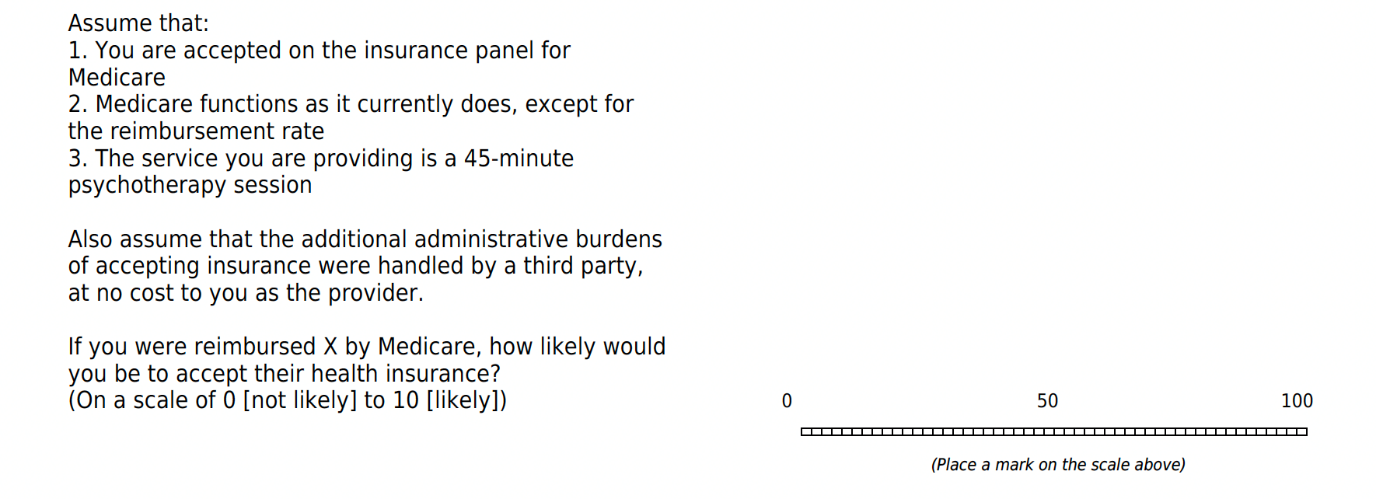
Page 22 (X is their current Medicare rate) **
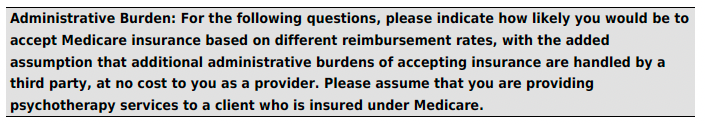
**

*Pages 23-33 are identical to Page 22 but with percentage increases in reimbursement rate as detailed in the methods*
